# A Glucosylated BODIPY Uses the GLUT Channel to Target Cancer Cells in *In Vitro* and *In Vivo* Models

**DOI:** 10.1021/acsomega.5c06410

**Published:** 2025-11-07

**Authors:** Marta Turati, Giacomo Biagiotti, Cosetta Ravelli, Chiara Tobia, Jacopo Cardellini, Luca Mignani, Jacopo Tricomi, Debora Berti, Stefano Cicchi, Barbara Richichi, Roberto Ronca

**Affiliations:** † Department of Molecular and Translational Medicine, 9297 University of Brescia, Brescia 25121, Italy; ‡ Department of Chemistry “Ugo Schiff”, 97031 University of Firenze, Via della Lastruccia 13, Sesto Fiorentino, Fi 50019, Italy

## Abstract

The conjugation of fluorescent probes to tumor-targeting molecules represents a promising strategy for the development of precision cancer bioimaging and treatment. Among the different tumor-targeting strategies, the use of d-glucose residues, which exploit the high energy demand of cancer cells, can enable recognition by a broad spectrum of tumors, thus overcoming limitations related to cancer heterogeneity. In this study, we combined the distinctive optical properties of BODIPY-based probes with the known tumor-targeting abilities of d-glucose. We report on the characterization of a glucosylated BODIPY, named **Glc-BODIPY**, and its ability to target different cancer cell types in both *in vitro* and *in vivo* models.

## Graphical abstract

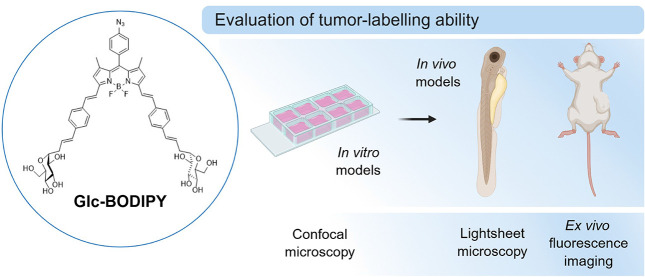

## Introduction

The design of fluorescent probes that can selectively target solid tumors and distinguish them from healthy tissues is of great interest for both surgery and diagnostic imaging.^1,2^ Over the years, several synthetic approaches have been developed for the conjugation of fluorescent probes to specific targeting molecules that recognize surface receptors commonly overexpressed in cancer cells. Despite such a huge amount of work, the applications of these conjugates are limited to specific tumor cells that express the molecular target.^3–5^ To the best of our knowledge, only a few examples of probes have shown broad applicability across diverse tumor settings, *i.e.*, cyanine^1,6^ and porphyrin derivatives.^7^ These probes have demonstrated active tumor-targeting capabilities and highlighted the need for further research in this field.

A common feature in cancer metabolism is the increased uptake and consumption of glucose due to the so-called Warburg effect, which enables cancer cells to meet the increased energetic demand associated with their uncontrolled proliferation.^8,9^ Accordingly, glucose transporters (GLUTs), together with key enzymes involved in glucose metabolism (e.g., hexokinase 2),^10^ have been observed to be markedly overexpressed in tumors in response to hypoxia-dependent HIF-1 transcriptional activity, as well as to a variety of oncogenes and growth factors.^11,12^ High GLUT expression levels have been described in several cancer types, including breast, lung, prostate, and colorectal carcinoma, positively correlating with tumor progression.^13,14^ Thus, approaches that exploit the “addiction” of tumor cells to glucose may enable the discrimination of malignant cells from normal ones and may represent an appealing strategy for both diagnostic and therapeutic purposes. For instance, the [^18^F]-2-deoxy-2-fluoro-d-glucose positron emission tomography (FDG-PET), a noninvasive diagnostic technique routinely employed in clinical practice, relies on the preferential tumor accumulation of a radiolabeled glucose analogue and represents clear evidence of the reliability of such an approach.^15,16^ Moreover, glucose conjugation has been exploited to preferentially deliver different therapeutic agents to cancer cells, such as photosensitive compounds for photodynamic therapy^17,18^ and radiopharmaceuticals.^19^ Similarly, both organic^20^ and inorganic^21,22^ glucose-bearing nanoparticles have been reported to display better uptake by cancer cells when compared to normal ones.^23^ Concerning fluorescent probe glycoconjugates,^2^ a glucosylated dual-modal imaging probe named 2-[^18^F]­FBDG has been recently described.^24^ It showed GLUT-dependent PET and fluorescence imaging capabilities useful for cancer diagnosis. However, *in vivo* radioactive defluorination was observed, raising concerns about its *in vivo* stability.

Since their discovery,^25^ 4,4-difluoro-4-bora-3a,4a-diaza-s-indacene (BODIPY) has showed huge potential and captured the interest of chemists and biologists due to its unique optical properties and chemical flexibility. The modularity of the BODIPY scaffold enables optimization for various bioimaging techniques and ensures compatibility with different fluorescence microscopy and detection instruments.

In this context, we have recently reported the synthesis of a highly fluorescent and red-emitting BODIPY, the **Glc-BODIPY** (Figure 1), which contains two metabolically stable d-glucose residues at the C-3 and C-5 positions of the BODIPY core.^26^ This probe showed excellent optical properties that allow for its use in advanced optical imaging techniques. Accordingly, we decided to move a step forward in the evaluation of its applicability in biological bioimaging settings. In particular, in this work, we investigated, both in *in vitro* and *in vivo* models, how the presence of the d-glucose residues provides a tumor-labeling ability to the **Glc-BODIPY** (Figure 1).

**Figure 1:**
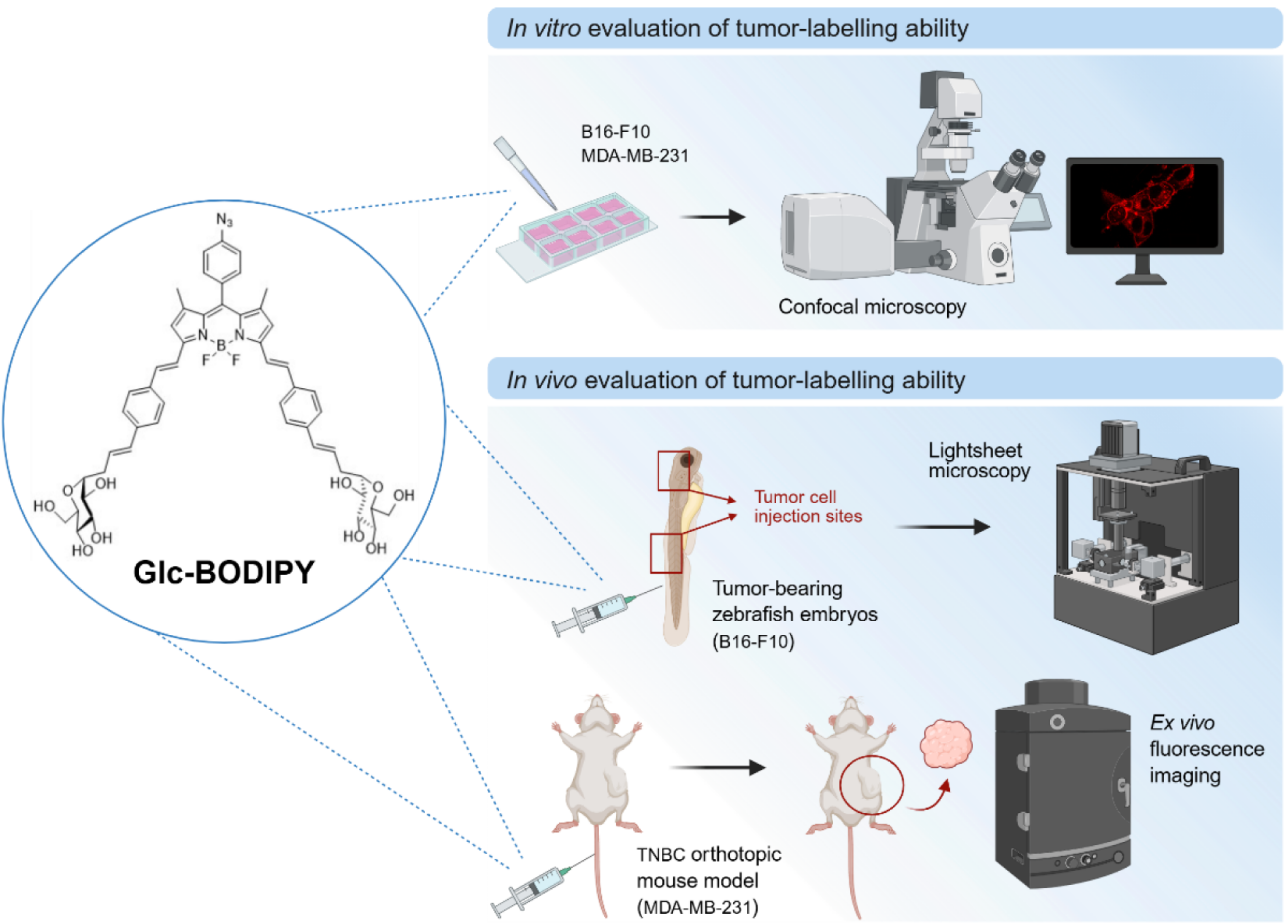
Schematic representation of the experimental workflow for the evaluation of the tumor-labeling ability of **Glc-BODIPY** both *in vitro* and *in vivo*. Created in BioRender. Ronca, R. (2025) https://BioRender.com/lx123eg.

## Results and Discussion

### d-Glucose Conjugation Ensures Tumor-Labeling Ability of the **Glc-BODIPY** *In Vitro*

**Glc-BODIPY** (Figure 2A) was synthesized following a previously reported protocol.^26^ First, the optical and physicochemical properties under experimental conditions for this study were assessed (see ESI, Figures S1–S2). The UV–vis spectra of **Glc-BODIPY** solutions (0.3% DMSO in water) showed a similar absorption profile to that previously reported by us in various organic solvents.^26^ However, upon excitation at 580 nm, the expected fluorescence emission band around 660 nm was scarcely observed (see ESI, Figure S1B). To investigate this behavior, dynamic light scattering (DLS) measurements were performed to assess the potential formation of nano-micrometric aggregates in solution. Figure S2 (see ESI) shows the measured autocorrelation functions of a 10 μM solution of **Glc-BODIPY**. No aggregate formation could be detected, as indicated by the absence of a defined correlation function. However, if Triton X-100, a nonionic surfactant, is added to the solution of **Glc-BODIPY** (0.3% DMSO in water), the fluorescence emission was restored (see ESI, Figure S1B). These data support the hypothesis that fluorescence quenching can be related to the formation of **Glc-BODIPY** oligomers, driven by π-π stacking interactions, whose size cannot be detected with DLS.

**Figure 2:**
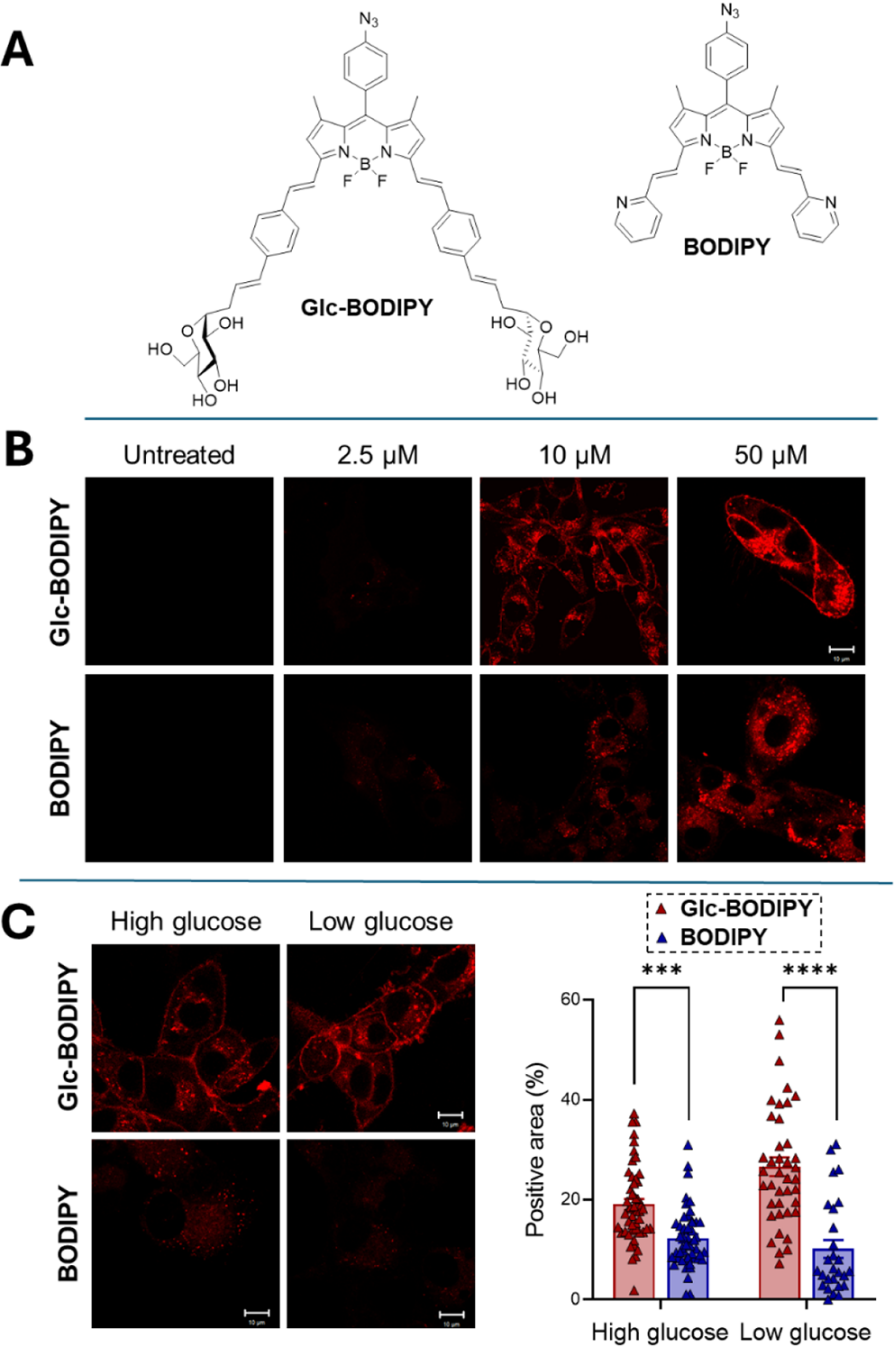
(A) Structures of **Glc-BODIPY** and **BODIPY.** (B) Representative images of B16-F10 cells incubated with increasing concentrations (0 (Untreated), 2.5, 10, 50 μM in DMEM) of **Glc-BODIPY** or control **BODIPY** in high glucose conditions. (C) B16-F10 cells incubated with a solution (10 μM in DMEM) of **Glc-BODIPY** or control **BODIPY** in high and low glucose conditions (left panel) and relative quantification (right panel). ***p < 0.001.

Then, we decided to move forward and evaluate the optical properties of the probe in cell settings. Accordingly, for the *in vitro* studies, two prototypic cell lines representing extremely aggressive and metastatic tumor models were selected: the murine melanoma B16-F10 cells and human triple-negative breast cancer (TNBC) MDA-MB-231 cells. A previously reported **BODIPY** ^27^ (Figure 2A) that shows similar optical properties compared to the **Glc-BODIPY,** was used as a negative control. It contains two pyridine residues instead of the d-glucose residues; thus, it can be useful to assess the effect of the sugar heads on the ability of **Glc-BODIPY** to recognize and label cancer cells. A dose-finding experiment was conducted by incubating B16-F10 cells for 15 min with **Glc-BODIPY** or with the **BODIPY** at different concentrations (0, 2.5, 10, 50 μM in DMEM) in high-glucose conditions (25 mM). The tumor cell-binding ability of the two probes was assessed by confocal microscopy and image quantification. As shown in Figure 2B, a low dose (2.5 μM) resulted in no signal in both experimental conditions, while the highest dose (50 μM) resulted in a strong fluorescent signal, mainly localized on the plasma membrane of B16-F10 cells treated with **Glc-BODIPY**, and in an almost equal but nonspecific/intracellular signal in cells incubated with **BODIPY**. Interestingly, at a dose of 10 μM, a significantly stronger fluorescent signal was observed in cells treated with **Glc-BODIPY** compared to those incubated with control **BODIPY**. The fluorescent signal was mainly confined to the plasma membrane, suggesting a specific binding conferred by the glucose residues. For these reasons, the 10 μM dose was selected for the following experiments.

To evaluate whether glucose concentration can influence the labeling of the cancer cell plasma membrane by **Glc-BODIPY**, B16-F10 cells were incubated with **Glc-BODIPY** or with control **BODIPY** for 15 min in both high and low (25 and 5.5 mM, respectively) glucose medium (Figure 2C). As expected, a significantly higher fluorescence signal was detected in cancer cells treated with **Glc-BODIPY** under both conditions when compared to cells incubated with control **BODIPY**. Notably, this difference was exacerbated when cells were treated in low glucose medium, consistent with the well-known upregulation of GLUT expression in response to low glucose concentration.^28,29^

These data suggest that glucose functionalization in **Glc-BODIPY** confers the capacity to recognize and label cancer cells, in comparison with **BODIPY**, which was used as a control and displays an extremely low targeting capability. Since the tumor-labeling property of **Glc-BODIPY** is further increased in low-glucose conditions, this setting was selected for the following experiments.

### The Tumor-Labeling Ability of **Glc-BODIPY** Depends on Interaction with GLUT Channels

To confirm the involvement of GLUT channels in the tumor-labeling ability of **Glc-BODIPY**, a competition assay was performed using WZB117, a GLUT-1 inhibitor^30^ capable of controlling the biological activity of the GLUT channel by inhibiting glucose transport inside the cells.^31^ B16-F10 cells were pretreated for 30 min with WZB117 (10 μM) and then incubated with **Glc-BODIPY** (10 μM) or control **BODIPY** (10 μM) for 15 min. As reported in Figure 3, a significant decrease in the **Glc-BODIPY** fluorescent signal was observed in B16-F10 cells pretreated with WZB117 compared to untreated cells. Notably, no difference was detectable in the fluorescence signal of cancer cells treated with control **BODIPY** in the presence or absence of WZB117 pretreatment.

**Figure 3:**
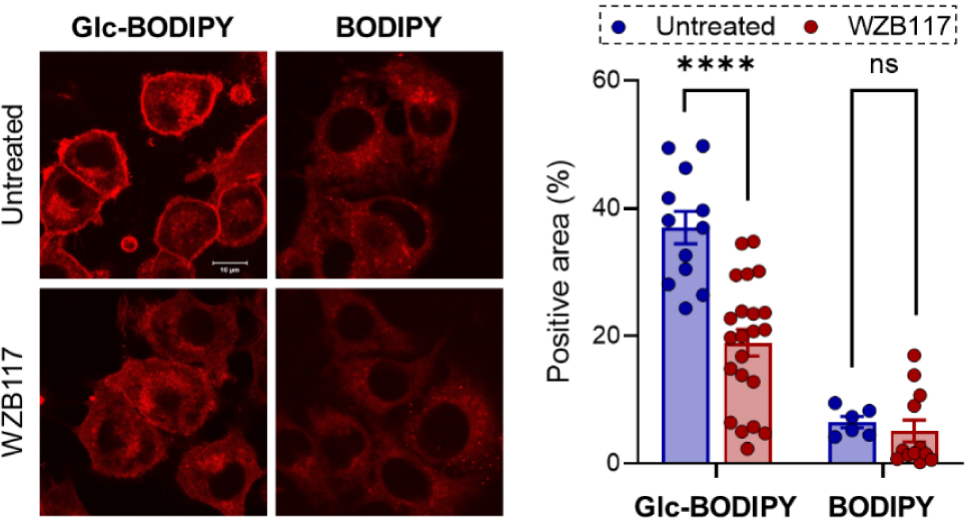
Representative images of B16-F10 cells incubated with a solution (10 μM) of **Glc-BODIPY** or control **BODIPY** in low glucose conditions in the absence or presence of WZB117 (left panel) and relative quantification (right panel). ****p < 0.0001.

These observations confirmed that the tumor-labeling ability displayed by **Glc-BODIPY** relies on its ability to interact with GLUT channels, which are frequently overexpressed on the membranes of various cancer cells.^32^

### *In Vitro* Tumor-Labeling Ability of **Glc-BODIPY** on Human TNBC Cells

What was observed in B16-F10 cells was also confirmed in a prototypic human TNBC cell line. MDA-MB-231 cells were incubated with increasing doses of **Glc-BODIPY** (1, 5, and 10 μM) for 15 min in the absence or presence of the WZB117 inhibitor (10 μM). As shown in Figure 4, **Glc-BODIPY** stains tumor cells in a dose-dependent manner with a clear membrane-localized signal. The concentration of 10 μM was found to be the most effective for the specific labeling of MDA-MB-231 cells. Moreover, in line with what was observed in B16-F10 cells, pretreatment with WZB117 resulted in a significant reduction of the fluorescent signal of **Glc-BODIPY**. Notably, the treatment of MDA-MB-231 cells with the control **BODIPY** resulted in faint and non-membrane-specific staining, which was not diminished upon treatment with WZB117.

**Figure 4:**
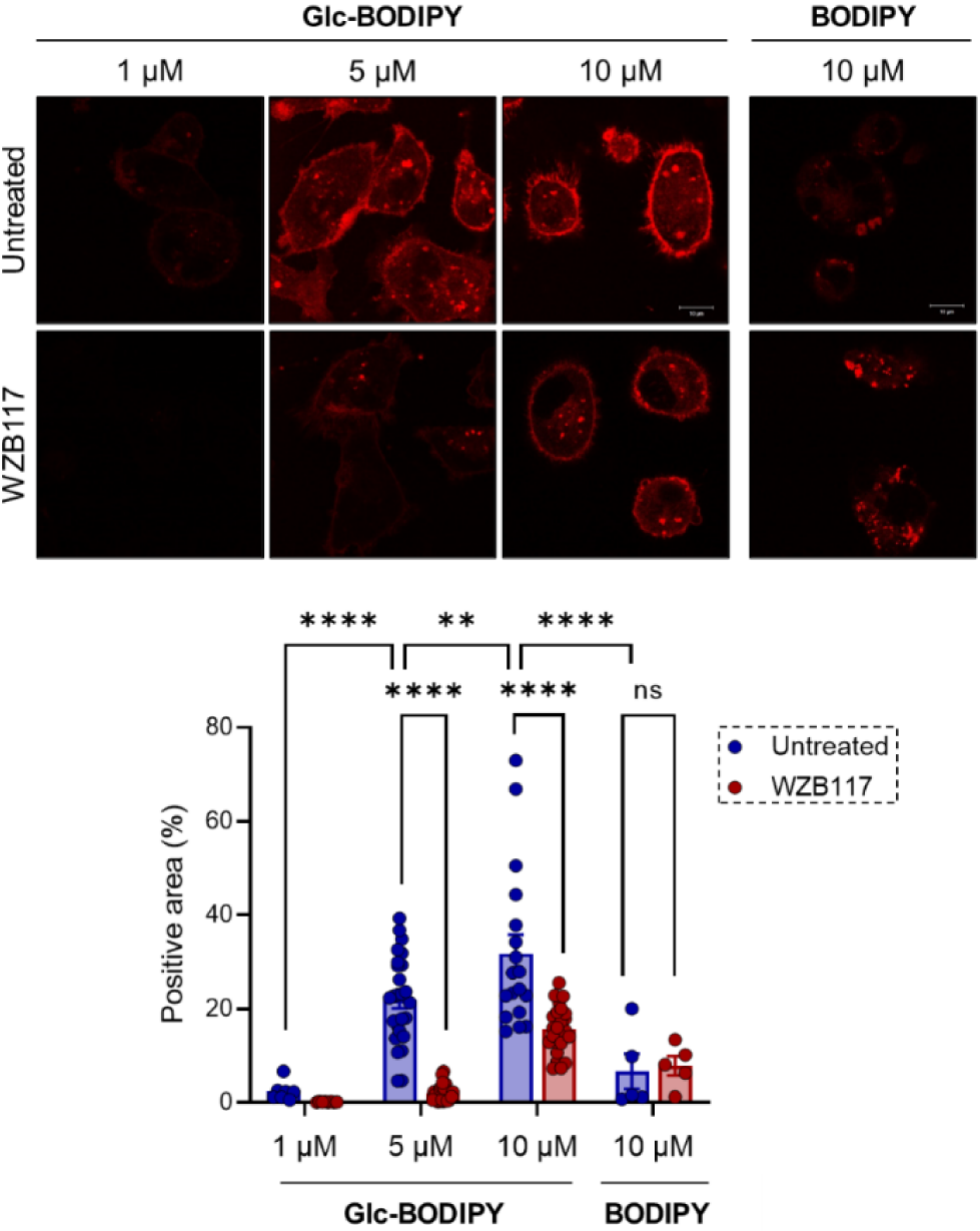
Representative images (upper panel) of MDA-MB-231 cells incubated with increasing concentrations of **Glc-BODIPY** (1, 5, 10 μM solutions in DMEM) or control **BODIPY** (10 μM) in low glucose conditions and in the absence or presence of WZB117 (10 μM), and relative quantification (lower panel). **p < 0.01; ****p < 0.0001.

These data, along with results obtained from previous *in vitro* experiments, confirm that **Glc-BODIPY** efficiently recognizes and labels both murine and human cancer cells, and that this interaction is blocked by the use of WZB117.^30^

### *In Vivo* Evaluation of the Tumor-Labeling Ability of **Glc-BODIPY**

In a translational perspective, the ability of **Glc-BODIPY** to recognize and label cancer cells was evaluated *in vivo* by exploiting two animal models represented by the zebrafish embryo and a TNBC orthotopic model. First, B16-F10 cells were injected into the medulla oblongata or the trunk of transgenic Tg­(*neurod1*:EGFP)^ia50^ zebrafish embryos 48 h post-fertilization (hpf) (Figure 5A). One hour later, 4 nL of a 10 μM solution of **Glc-BODIPY** or control **BODIPY** was injected into tumor-bearing zebrafish embryos through microangiography, and imaging analysis was performed the following day at 72 hpf. In line with what was observed *in vitro,* a higher fluorescent signal was detected in correspondence with cancer cells inoculated into the medulla oblongata of zebrafish embryos injected with **Glc-BODIPY** when compared to control **BODIPY** treatment, as shown both in the 3D rendering (Figure 5B) and in the maximum intensity projection (MIP) of selected planes from light sheet images (Figure 5C). Of note, the fluorescent signal of **Glc-BODIPY** is mainly localized in the outer cell membrane of cells injected into zebrafish embryos, as shown in Figure 5C at higher magnification. In line with these observations, a fluorescent signal was detected in correspondence with cells injected into the trunk of zebrafish embryos treated with **Glc-BODIPY**, while only a faint signal was detected in embryos injected with control **BODIPY** (Figure 5D). Of note, **Glc-BODIPY** displays a nonspecific fluorescent signal deriving from host tissues when injected into the medulla oblongata but not into the trunk of zebrafish embryos.

**Figure 5:**
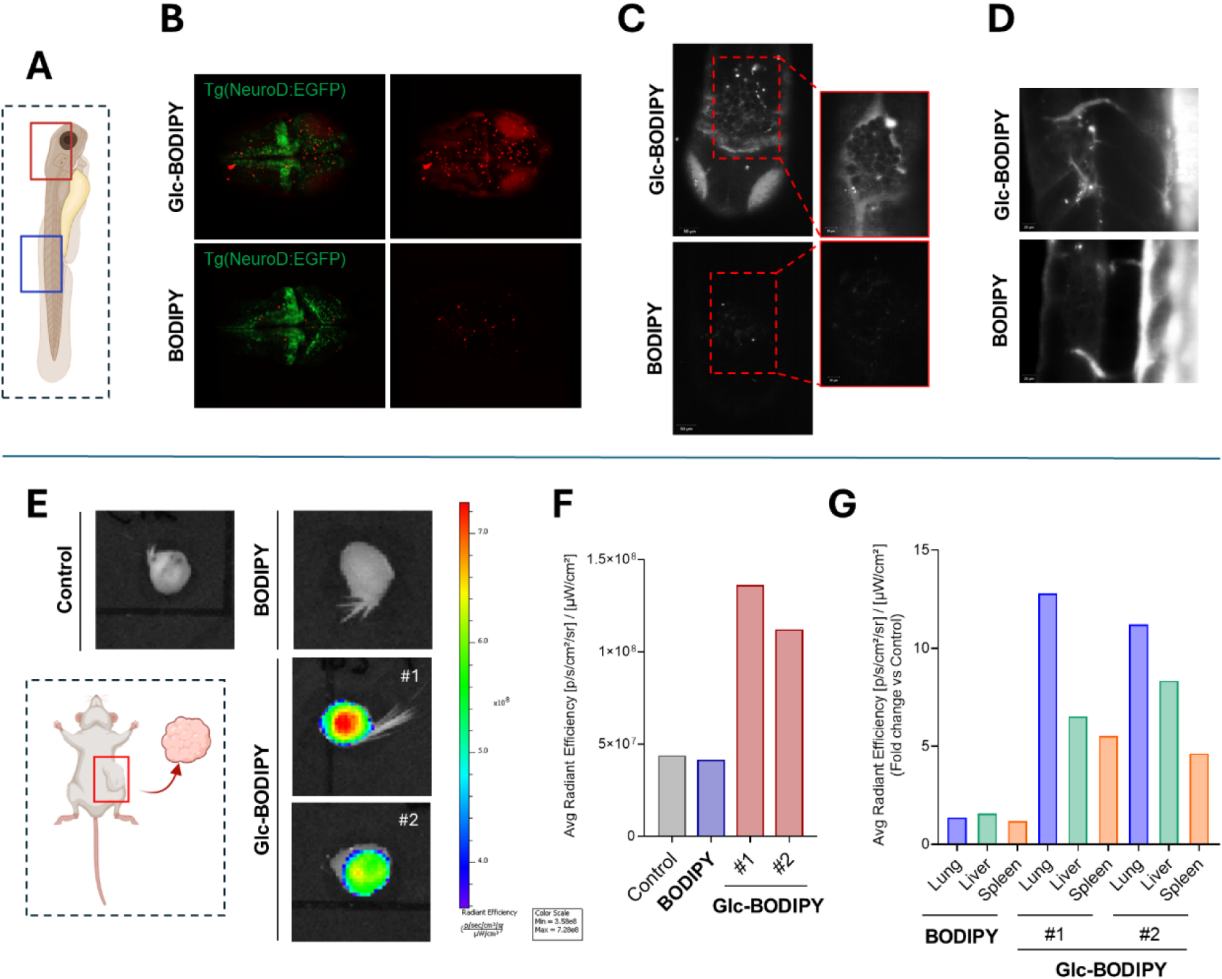
(A) Schematic representation of the B16-F10 injection site in the zebrafish (medulla oblongata in red and trunk in blue). (B) 3D rendering of the head of tumor-bearing live embryos, dorsal view, anterior to the right. (C) MIP of selected planes from Light Sheet images of fixed tumor-bearing embryos treated with **Glc-BODIPY** or control **BODIPY**, dorsal view, anterior to the bottom (left panel), and magnification of the tumor mass in the medulla oblongata (right panel). (D) MIP of selected planes from Light Sheet images of fixed tumor-bearing embryos treated with **Glc-BODIPY** or control **BODIPY**, lateral view, anterior to the top. (E) Bioluminescence imaging of MDA-MB-231 tumors harvested from mice untreated (Control) (*n* = 1) or treated with **Glc-BODIPY** (20 μM solution in PBS-20% DMSO; 150 μL/mouse) (*n* = 2) or control **BODIPY** (*n* = 1) and relative quantification (F). (G) *Ex vivo* bioluminescent quantification (expressed as fold change relative to the untreated control) of lungs, liver, and spleen collected from mice treated with **Glc-BODIPY** or control **BODIPY** (Created in BioRender.com).

Furthermore, the tumor-targeting properties of **Glc-BODIPY** were evaluated in an orthotopic xenograft mouse model. In particular, human MDA-MB-231 cells were inoculated into the mammary fat pad of immunocompromised mice, and **Glc-BODIPY** or the control **BODIPY** was injected intravenously once tumors were palpable. Twenty-four hours after injection, tumors (Figure 5E) and organs (i.e., lungs, liver, and spleen) (Figure 5G) were harvested, and *ex vivo* fluorescence imaging was performed. A fluorescent signal was detected only in tumors collected from mice injected with **Glc-BODIPY,** whereas control **BODIPY** displayed a fluorescent signal comparable to that of tumors collected from untreated mice (Figure 5F). These data are in line with the *in vitro* and *in vivo* analyses on zebrafish embryos described above. Additionally, the *ex vivo* imaging of explanted organs showed a fluorescent signal of the **Glc-BODIPY** in the lungs, liver, and spleen of treated mice (Figure 5G). Conversely, limited probe accumulation in the other organs was observed in the mice treated with control **BODIPY** (Figure 5G), indicating fast clearance/metabolism of the probe. These data are in line with seminal studies on the biodistribution of the FDG-PET probe, which clearly demonstrate that animal handling significantly affects the biodistribution of compounds that exploit glucose metabolism. Specific conditions are required to reduce the off-target distribution of the probe, thereby making the observation of its specific tumor accumulation in *in vivo* models challenging.^33,34^ Accordingly, our findings altogether support the hypothesis of the tumor-labeling ability of **Glc-BODIPY**.

## Conclusions

The conjugation of fluorescent probes to tumor-targeting molecules represents a compelling strategy for discriminating malignant cells from healthy tissues. Unlike targeting surface receptor overexpression or genetic alterations that do not occur in all cancer types, exploiting cancer-associated metabolic alterations, which are more common among cancer subtypes and across cancers, enables us to selectively visualize a broad spectrum of tumor types. Among the metabolic changes associated with tumor cells, the increase in glucose metabolism and glucose dependence is well described and it has already been exploited in clinical practice for FDG-PET.

In this work, we have characterized the tumor-labeling ability of a glucose-functionalized BODIPY by exploiting both *in vitro* and *in vivo* models of melanoma and TNBC. We observed that incubation with **Glc-BODIPY** *in vitro* correlates with a strong membrane fluorescent signal in both cancer cell types, which is strongly dependent on the interaction between the glucose moiety and GLUTs, frequently overexpressed on the membranes of cancer cells. The tumor-targeting ability of **Glc-BODIPY** was further validated *in vivo* by exploiting both ectopic and orthotopic models in zebrafish and mice, respectively.

The development of novel tumor-targeting fluorescent probes is of great interest for both surgical applications and diagnostic bioimaging. Potentially, fluorescent probes could find applications in highlighting the tumor mass and serving as an aid to surgeons by simplifying the identification of the tumor margin and enabling precise resection of the mass and/or identification of microlesions.^35^ Moreover, over the past decade, BODIPY derivatives have demonstrated excellent potential as photosensitizing agents, enabling their use in photodynamic therapy, an emerging approach for local, controllable, and noninvasive cancer treatment.^36^ Hence, tumor-labeling BODIPY derivatives represent promising candidates for both cancer bioimaging and treatment.

## Materials and Methods

### Cell Culture

Murine melanoma B16-F10 cells were maintained in RPMI medium supplemented with 10% fetal bovine serum (FBS). Human TNBC MDA-MB-231 cells were grown in Dulbecco’s Modified Eagle’s Medium (DMEM) containing 10% FBS. Cells were maintained in an incubator at 37 °C in a humidified atmosphere containing 5% CO_2_.

### Live Cell Staining and Imaging

For dose-finding experiments, 40000 B16-F10 and MDA-MB-231 cells were seeded in 8-well Nunc Lab-Tek II chambered coverglass, and after 24 h, they were treated with **Glc-BODIPY** and control **BODIPY** at increasing concentrations (2.5, 10, 50 μM for B16-F10 and 1, 5, 10 μM for MDA-MB-231 cells) in high and low glucose medium (25 and 5.5 mM, respectively) for 15 min. The impact of glucose concentration on the tumor-labeling ability of the two probes was assessed by incubating B16-F10 cells with 10 μM **Glc-BODIPY** and control **BODIPY** in high or low glucose conditions for 15 min. After incubation, cells were washed with phosphate-buffered saline and placed in medium (Hank’s balanced salt solution) without phenol red. The tumor-labeling ability of **Glc-BODIPY** and control **BODIPY** was assessed in live cells using an LSM900 confocal microscope equipped with a Plan–Apochromat 63*x*/1.4 Oil objective (Carl Zeiss, Oberkochen, Germany). **BODIPYs** were excited with a 543 nm laser and acquired at 633–695 nm.

### Competition Assay

40000 B16-F10 and MDA-MB-231 cells were seeded as described, and after 24 h, they were pretreated for 30 min with 10 μM WZB117 (MedChemExpress, CAS 1223397-11-2). Subsequently, the cells were incubated with **Glc-BODIPY** (10 μM for B16-F10 and 1, 5, or 10 μM for MDA-MB-231 cells) or with 10 μM control **BODIPY** for 15 min under low-glucose conditions. The cells were then washed, and the tumor-binding activity was assessed through fluorescence microscopy.

#### *In Vivo* Studies

##### Zebrafish Model

Zebrafish embryos were handled according to relevant national and international guidelines. Current Italian regulations do not require approval for research on zebrafish embryos. Zebrafish were raised and maintained under standard laboratory conditions as described.^37^ Briefly, the transgenic reporter Tg­(*neurod1*:EGFP)^ia50^ line was maintained at 28 °C under a 14 h light/10 h dark cycle. Immediately after spawning, the fertilized eggs were harvested, washed, and placed in 10 cm Petri dishes in fish water. Embryos were incubated at 28 °C and staged as described.^38^ B16-F10 cells were injected into the medulla oblongata (200 cells per embryo) or into the trunk (100 cells per embryo) of 48 hpf transgenic Tg­(*neurod1*:EGFP)^ia50^ zebrafish embryos. One hour later, 4 nL of 10 μM **Glc-BODIPY** or control **BODIPY** were injected through microangiography, and imaging analysis was performed the following day at 72 hpf using a lightsheet microscope. 3D rendering of live embryos was performed using Arivis Software.

##### Mouse Model

Animal experiments were performed in accordance with Italian laws (D.L. 116/92 and subsequent amendments) that enforce the EU 86/109 Directive and were approved by the local animal ethics committee (OPBA, Organismo Preposto al Benessere degli Animali, Università degli Studi di Brescia, Italy). MDA-MB-231 cells (3 × 10^6^) were inoculated into the mammary fat pad of immunocompromised NOD/SCID female mice. Once tumors became palpable, **Glc-BODIPY** or the control **BODIPY** was injected intravenously (150 μL/mouse of a 20 μM solution prepared in PBS-20% DMSO), and after 24 h, tumors and organs (i.e., lung, liver, and spleen) were harvested, and *ex vivo* fluorescence imaging was performed using IVIS Lumina III (Revvity).

## Supplementary Material



## Data Availability

The data underlying this study are available in the published article and its Supporting Information.

## Notes

Materials and methods for the optical and chemical-physical characterization of **Glc-BODIPY** have been provided (PDF)
