# Supplementary material for: A Glucosylated BODIPY Uses the GLUT Channel to Target Cancer Cells in In Vitro and In Vivo Models
Source: ACS Omega. 2025 Nov 7;10(45):54217–23. doi: 10.1021/acsomega.5c06410 (PMC12631688; doi:10.1021/acsomega.5c06410)
Supplement: Supplementary file 1 [file ao5c06410_si_001.pdf]

Supporting Information

A glucosylated BODIPY uses the GLUT channel to target cancer cells in *in vitro* and *in vivo* models

Marta Turati,[a] Giacomo Biagiotti,[b] Cosetta Ravelli,[a] Chiara Tobia,[a] Jacopo Cardellini,[b] Luca Mignani,[a] Jacopo Tricomi,[b] Debora Berti,[b] Stefano Cicchi,[b] Barbara Richichi,[*,b] Roberto Ronca[*,a]

[a.] *Department of Molecular and Translational Medicine, University of Brescia, Brescia, Italy.*

E-mail: roberto.ronca@unibs.it

[b.] *Department of Chemistry "Ugo Schiff", University of Firenze, Via della Lastruccia 13, 50019 Sesto Fiorentino, Fi, Italy.*

E-mail: barbara.richichi@unifi.it

Table of content

Figure S1 pg S3

Figure S2 pg S3

Sample preparation pg S3

Optical characterization of Glc-BODIPY pgS4

Methods for dynamic light scattering pg S4

References pg S4

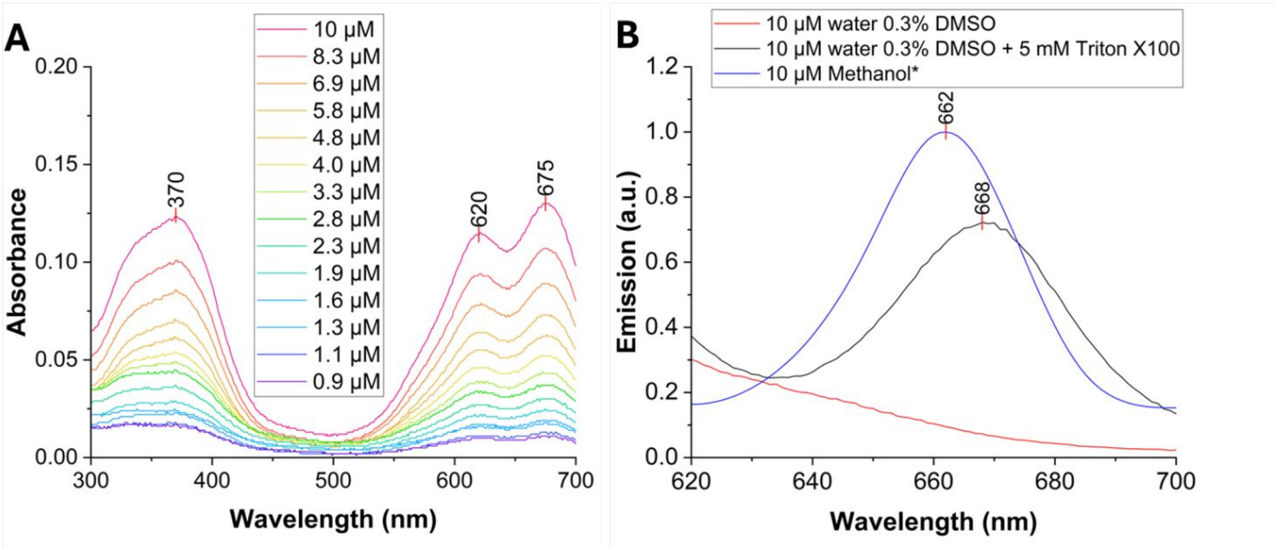

Figure S1. A) UV-Vis absorption spectra of **Glc-BODIPY** solutions in water + 0.3% DMSO; B) Emission spectra of a solution of **Glc-BODIPY** (10 μM in methanol**,** 10 μM in water + 0.3 % DMSO and 10 μM in water + 0.3 % DMSO + 5 mM Triton X100). *UV-vis data in Methanol were previously reported. [1]

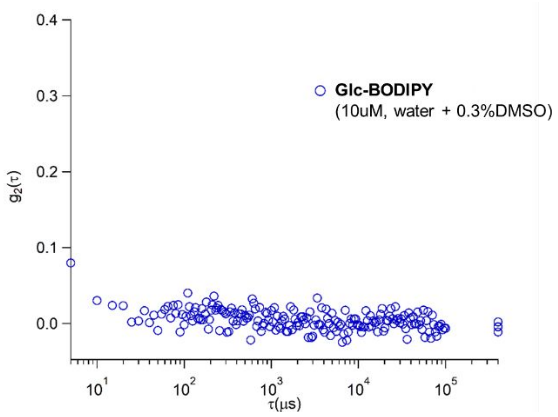

Figure S2. Dynamic light scattering autocorrelation functions of a solution of **Glc-BODIPY** (10 μM in water + 0.3% of DMSO).

Sample preparation. The solutions of **Glc-BODIPY** in water + 0.3% or + 20% DMSO were prepared as follow: 0.8 μL of a 3.12 mM solution of **Glc-BODIPY** in DMSO was diluted with 250 μL of water or desired buffer to afford the 10 μM 0.3 % DMSO solution; a 100 μM solution of a 3.12 mM solution of **Glc-BODIPY** in DMSO was diluted 1:5 in water or desired buffer to afford the 20 μM 20% DMSO solution.

Optical characterization of Glc-BODIPY. UV–vis spectra were recorded on a BMG Labtech SPECTROstar Nano UV–vis spectrophotometer using a 1 cm cell. Fluorescence spectra were registered on a HORIBA FluorMax® Plus spectrofluorimeter using 1.0 cm cell.

Methods for dynamic light scattering. Dynamic light scattering (DLS) measurements were performed using a Brookhaven Instruments setup, comprising a BI 9000AT correlator and a BI 200 SM goniometer. The signal was detected by an EMI 9863B/350 photomultiplier. The light source was the second harmonic of a diode Nd:YAG laser, λ = 532 nm, Coherent DPY315M-100, linearly polarized in the vertical direction. The autocorrelation functions of the scattered light were measured at 90 and 25 °C.

References

1 G. Biagiotti, E. Purić, I. Urbančič, A. Krišelj, M. Weiss, J. Mravljak, C. Gellini, L. Lay, F. Chiodo, M. Anderluh, S. Cicchi and B. Richichi, Combining cross-coupling reaction and Knoevenagel condensation in the synthesis of glyco-BODIPY probes for DC-SIGN super-resolution bioimaging, *Bioorg. Chem.*, 2021, **109**, 104730.
